# Improving the Technique of Pelvic Floor Muscle Contraction in Active Nulliparous Women Attending a Structured High–Low Impact Aerobics Program—A Randomized Control Trial

**DOI:** 10.3390/ijerph19105911

**Published:** 2022-05-12

**Authors:** Magdalena Piernicka, Monika Błudnicka, Damian Bojar, Jakub Kortas, Anna Szumilewicz

**Affiliations:** 1 Department of Fitness, Gdansk University of Physical Education and Sport, 80-336 Gdańsk, Poland; anna.szumilewicz@awf.gda.pl; 2 Department of Clinical Physiotherapy and Professional Practices, Gdansk University of Physical Education and Sport, 80-336 Gdańsk, Poland; mbludnicka@interia.pl; 3 Department of Sport Science, Gdansk University of Physical Education and Sport, 80-336 Gdańsk, Poland; bojar.damian.db@gmail.com; 4 Department of Biomechanics and Sports Engineering, Gdansk University of Physical Education and Sport, 80-336 Gdańsk, Poland; jakub.kortas@awf.gda.pl

**Keywords:** Kegel exercises, muscle onset, exercise professionals, pelvic floor muscle contraction, high impact activity, electromyography, urinary incontinence

## Abstract

Learning the correct technique of performing pelvic floor muscle (PFM) exercises is a very important factor influencing the effectiveness of this muscle group training. Correctly performed PFM contractions are involved in the urinary continence mechanism. In this study, we tested the hypothesis that a six-week high-low impact aerobics program, supported by one EMG biofeedback session and pelvic floor muscle training, improves the technique of PFM contraction. Participants were 42 active nulliparous women (age 22 ± 2 years, mean ± SD), randomly allocated into intervention (*n* = 18) and control (*n* = 24) groups. We analyzed the technique of PFM contractions, taking into account the order in which selected muscle groups were activated, so called ‘firing order’. In both groups, we assessed the PFM contraction technique using surface electromyography (sEMG) and intravaginal probes, before and after six weeks of intervention. The intervention group received one biofeedback session on how to properly contract PFM and afterwards participated in a high-low impact aerobics program supplemented by PFM training. The control group did not receive any intervention. In the pre-test, 67% of the intervention group activated PFM first in order in short, quick contractions. After six weeks of training, this task was correctly performed by 100% of this group (*p* = 0.04). The proper performance of PFM short contraction in the control group was 75% and 67%, before and after intervention, respectively. In the intervention group we also observed statistically significant improvement in the PFM contraction technique in 10-s contractions. The presented intervention was beneficial for the improvement of PFM contraction. High–low impact aerobics, supplemented by one EMG biofeedback session and pelvic floor muscle training can be recommended for active nulliparous women.

## 1. Introduction

Pelvic floor muscle training is recommended as a preventive [1,2] and curative treatment of the symptoms of lower urinary tract dysfunction in people of all ages. The main pelvic floor dysfunctions (PFD) that can be alleviated by introducing appropriate pelvic floor muscle training (PFMT) include: stress urinary incontinence; urinary and fecal incontinence [3,4]; sexual dysfunction [5]; and prolapse of the pelvic organs [6,7]. The symptoms of urinary incontinence (UI) are felt both mentally and physiologically [8]. Decreased quality of life caused by uncontrolled leakage of urine leads to emotional disturbances, reduced professional activity and social isolation [9]. Urinary system ailments worsen in situations where there is an increase in intra-abdominal pressure—most often during jumping, running, sneezing, coughing, laughing and lifting weights [9,10,11]. Learning and the ability to consciously use “the knack” while performing these activities may help prevent uncontrolled leakage of urine [10,11]. Pelvic floor muscle training refers to performing repetitive voluntary contractions of the pelvic floor muscles (PFM), following a protocol that determines the frequency, intensity, and type of exercise, as well as the duration of training. The proposed training programs typically include one or more sets of exercises per day, performed at least several days per week [4]. In order to ensure more lasting effects, it is recommended to continue the exercises in the longer term [12]. For people starting PFMT, a big problem is the correct location and isolation of the exercised muscle group [13]. The most common mistakes include contraction of other synergistic muscles (inter alia the external oblique muscle, the rectus abdominis muscle, and the gluteus maximus muscle) and holding breath [14]. Learning the correct movement patterns is essential for greater efficiency. Strong and fast PFM contractions are involved in the urinary continence mechanism [15] and therefore they should be regularly performed. The training process influencing the improvement of muscle function should include contractions of maximum strength, of various duration, and should include exercises developing coordination skills [16]. In this study, we aimed at testing the hypothesis that a six-week high-low impact aerobics program, supported by one EMG biofeedback session and pelvic floor muscle training, improves the technique of PFM contraction.

## 2. Materials and Methods

Forty two female students of the Gdansk University of Physical Education and Sport participated in the study. Participants aged 19 to 28 years (22 ± 2 years; mean ± SD) were randomly allocated in the intervention or control groups originally in a 1: 1 ratio. Before and after the experiment, we used the Incontinence Impact Questionnaire (IIQ) to evaluate the quality of life related to urinary incontinence [17]. We used the following criteria to assess quality of life: scores above 70 indicated poor quality of life; a score in the range of 50–70 indicated a moderate quality of life; scores below 50 were classified as a good quality of life. The applied IIQ scoring criteria were assigned based on a study by Corcos et al.: “Identifying cut-off scores with neural networks for interpretation of the incontinence impact questionnaire” [18]. Only the subjects with a good quality of life (IIQ < 50) were qualified to participate in the study.

**Figure 1:**
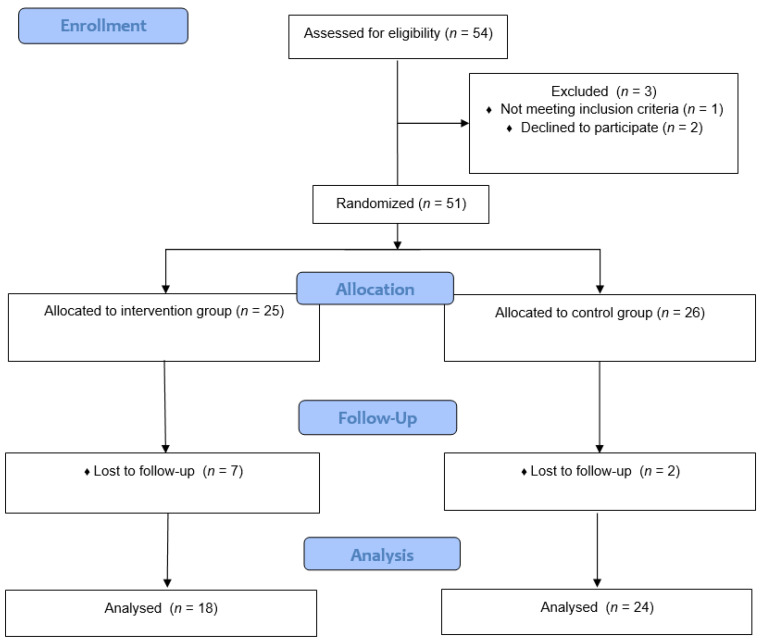
The flow of participants through the study.

Women with previous births, who were pregnant, or allergic to the materials used in the study and with contraindications to physical activity were excluded. Another qualification criterion for the study was the lack of diagnosed problems with the urinary tract. The process of qualifying participants for analysis is presented in Figure 1.

All assessments were carried out at the Laboratory of Physical Effort and Genetics in Sport at Gdansk University of Physical Education and Sport (GUPES) in Poland. We conducted all research interventions in accordance with the principles of the WMA Helsinki Declaration and with the consent of Bioethics. Commission at the Regional Medical Chamber in Gdansk (KB–8/14). Study participants signed their informed consent prior to testing. The research was carried out as part of the research project entitled: “Training of the pelvic floor muscles with surface electromyography” registered in the ISRCTN register under the number ISRCTN92265528, DOI 10.1186/ISRCTN92265528, run at GUPES in the years 2012–2018.

Before starting the intervention, all participants attended a lecture by a pelvic floor muscle expert on the importance of healthy pelvic floor function and its impact on women’s quality of life. During the educational session, the participants were informed about the correct technique of performing the PFM contraction. In both groups we assessed the technique of PFM contraction with surface electromyography (sEMG) using a vaginal probe (self-applied by the participant) and surface electrodes affixed to the gluteus maximus, rectus abdominis and external oblique muscles, before and after six weeks of the experiment. The vaginal probe used in the study was a Lifecare PR-02, Everyway Medical Instruments Co., Ltd., Taiwan. The PR-02 probe is lightweight (23.1 g) and it has a comfortable shape, which makes it easy to apply. The overall length is 76 mm and it is 28 mm in diameter. We used surface disk electrodes (SKINTACT Premier W-60, LEONHARD LANG GmbH, Innsbruck, Austria) for selected synergistic muscles [19].

To illustrate the correct implementation of the PFM contraction for the participants, we described contracting this muscle group as if they wanted to stop the flow of urine [20]. During the EMG assessment, the examined person assumed the following position: lying on their back, legs bent at the knees to an angle of 90 degrees, arms along the body. Women were reminded to breathe freely while exercising. During the individual PFM contraction, study participants were asked to completely relax the synergistic muscles. The PFM were assessed using the surface electromyography method based on the Glazer protocol [21]. The order of the individual motor tasks during a single test sequence was as follows: 10-s relaxation (baseline EMG of the pelvic floor muscle), one maximal short contraction (each contraction was followed by a return to muscle relaxation), five 10-s maximal contractions followed by 10-s relaxation followed by a 60-s contraction followed by 10-s relaxation. After performing the first sequence of EMG testing, the intervention group additionally repeated the entire sequence of exercises using the EMG biofeedback method. EMG biofeedback uses a vaginal probe to record the electrical activity of the PFM, which is displayed on the screen, making it a common clinical tool to enhance the effects of PFM training [22]. The use of this method is intended to facilitate the learning of the correct contraction technique and to remember these movement patterns by following the exercise program [22,23].

We used the MyoResearch (XP Master Edition 1.08.32) software to evaluate the technique of PFM contraction. The software assesses whether the selected muscles have been activated, and if so, in what order (so called ‘firing order’), based on a predetermined threshold for electromyographic activity. A muscle was considered active when muscle EMG activity exceeded three times the standard deviation of the baseline EMG value. In order to avoid disturbances caused by single spikes of neuromuscular activity in the PFM contraction, a duration of 0.2 s was used to keep the constant activity over the threshold [11]. The use of software that automatically instructs the study participants when to contract or relax PFM makes it possible to create the same test conditions for the whole group and to accurately determine the EMG firing order of selected muscles in PFM contraction [24]. When assessing the technique of PFM contraction, we took into account the firing order of the following muscles: the pelvic floor muscles; rectus abdominis muscle; the external oblique muscle; and the gluteus maximus muscle. The correct and highest-rated technique is to activate the PFM, keeping the synergistic muscles inactive. In this work, we analyzed the firing order of the pelvic floor muscles, using a scale of 1–5, as follows: (1) PFM activated first in order (the most beneficial performance of the pelvic floor muscle contraction); (2) PFM activated second; (3) PFM activated third; (4) PFM activated fourth in order; (5) lack of PFM neuromuscular activity. In order to classify the technique, we have used a scale of 4–1 taking into account the isolation of the PFM from the synergistic muscles. A maximum of four scores were assigned to the correct contraction technique, where PFM contraction was noted, maintaining the synergistic muscles relaxed. We assigned three scores to a technique, in which the PFM were activated first, and selected synergistic muscles later. Activating the synergistic muscles first followed by contraction of the PFM was scored as two points. We gave one point for synergistic muscle activity, leaving the PFM muscles relaxed [11].

Between the first and second sEMG assessments, the intervention group participated in a high–low impact aerobics training program supplemented with pelvic floor muscle training. The control group did not undergo any intervention. Before starting the training program, we assessed the exercise capacity of the women in the intervention group and their exercise heart rate (HR) ranges. We conducted cardiopulmonary exercise tests to exhaustion using an electronically regulated bicycle ergometer with a gas analyzer (Oxycon Pro, Erich JAEGER GmbH, Hoechberg, Germany) under laboratory conditions. A detailed description of the test method with the loading protocol used was presented by Kostrzewa-Nowak et al. [25] Before starting the experiment, the following performance parameters were recorded in the intervention group: VO2max (34.8 ± 6.3); HRmax (180 ± 15). A six-week training intervention was decided, based on the literature review. The duration of the training program was analyzed, after which the researchers noted the desired changes in neuromuscular activity of PFM [26]. During the high-low impact aerobics training unit, each woman exercised at an individual level of 60–75% HRmax determined on the basis of the cardiopulmonary assessments.

The study used a pelvic floor muscle training program based on ‘Graduated Strength Training’ aimed at shaping various motor skills [27]. Particular exercises and the number of sessions varied from week to week. In the first week, the women performed quick flicks of the pelvic floor muscles, learning to react quickly and control the contraction (five sets of ten repetitions). In the second week, the exercises involved more complete activation of the pelvic floor muscles by grading the contraction. A single exercise consisted of three increasingly stronger contractions of the pelvic floor muscles, followed by a return to full relaxation (five sets of ten repetitions). The goal of the third week exercise was endurance by keeping the pelvic floor muscles contracting for 10 s (three sets of ten repetitions). The next stage of the training focused on high intensity. One repetition of the exercise in the fourth week of the program consisted of performing three more and more intense contractions, maintaining the maximum contraction for 10 s and additionally prolonged with a short pulsation before returning to relaxation (three series of five repetitions). During the training in the fifth week, the intervention group performed a sequence of contractions and relaxation three times, identical to the sequence of EMG neuromuscular activity assessment [21]. The exercise was aimed at improving the speed, endurance and strength of the muscles, as well as consciously returning to relaxation. In the last week of the training program, the women repeated the set of exercises performed in the fourth week (two series of five repetitions). Additionally, in order to implement correct habits, the participants were asked to contract the pelvic floor muscles while performing household activities, e.g., lifting heavy objects [27]. The PFMT program was conducted by a qualified pelvic floor muscle training specialist.

Statistical analysis was performed using Statistica 13.1 software. Graphs were created by GraphPad Prism 7. All values are expressed as mean ± standard deviation (SD). The baseline differences between groups were analyzed by Mann–Whitney test. The response after the training program (pre-post measurements) in two groups (intervention and control) was evaluated by analysis of variance (ANOVA Friedman’s test). Dunn–Bonferroni post-hoc tests were performed to identify significantly different results within each group. Statistical significance was set at *p* < 0.05.

The sample size was predetermined by using a sample size calculation with the software G*power version 3.1.9.4. It was assumed that clinically relevant improvement in firing order of selected muscles in PFM contraction in the quick flick would be related to higher technique scores by 20%, following the training program. The estimation of the sample size showed that 22 participants should be included in each group (α = 0.05, β = 0.8). We added three people per group in order to allow for the possibility of loss.

## 3. Results

In Table 1 we present the characteristics of the study participants at baseline. The intervention and control groups did not differ significantly in terms of age, BMI and IIQ scores in all variables regarding the firing order of the pelvic floor muscles and the obtained technique scores of the pelvic floor muscle contraction in all selected motor tasks (Table 1).

After the exercise intervention, we reported statistically significant differences between groups in four variables: in the firing order of the pelvic floor muscles in the quick flick, in the first and in the fifth 10-s contractions (*p* = 0.04 for these three outcomes; Table 2), and also in the technique score presented in the quick flick (*p* = 0.03; Table 2).

**Figure 2:**
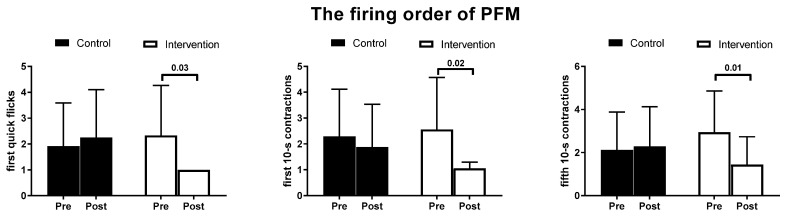
Changes in the firing order of the pelvic floor muscles in: (**a**) quick flick; (**b**) first 10-s contractions; (**c**) fifth 10-s contractions.

Based on the post hoc analysis, in the intervention group we observed a substantially beneficial change in the quick flick, both in the firing order and the technique score of the pelvic floor muscle contraction (*p* = 0.03 and *p* = 0.04, respectively; Table 2). In the post-intervention sEMG assessment, all women in the intervention group activated the PFM first in order in the quick flick. What is more, the intervention group statistically significantly improved the firing order of PFM in the first and in the fifth 10-s contractions (*p* = 0.02 and *p* = 0.01, respectively; Figure 2).

**Figure 3:**
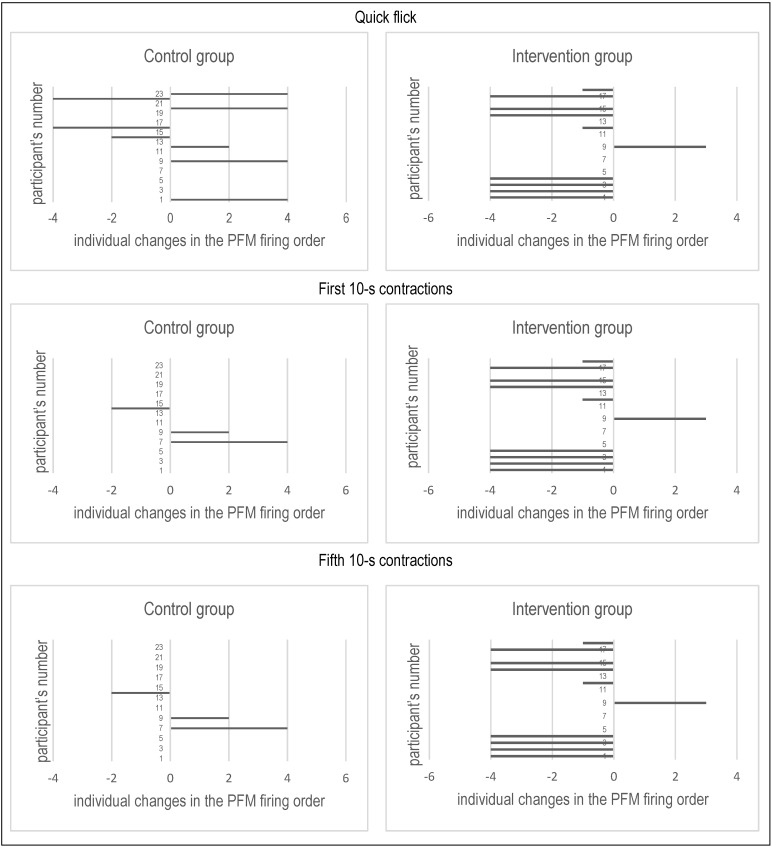
Individual changes in the firing order of pelvic floor muscles in selected motor tasks.

In this group, the changes in the firing order and in the technique score of PFM contraction in all other motor tasks, except for the technique score in the fourth 10-s contraction, also had beneficial tendency, but were not statistically significant. In the control group we reported no substantial changes in any of the analyzed variables. Figure 3 shows individual changes in the firing order of PFM in the intervention and control group. A positive result is the reduction in the individual value, which translates into earlier activation of PFM, and thus into a better technique of performed exercises (Figure 3).

Based on IIQ score, all women maintained good quality of life in terms of UI (Table 1). There was no deterioration in quality of life from participating in a high–low impact aerobics program. The intervention group after six-week training reported a reduction in the symptoms of uncontrolled urine leakage (pre 4.76 ± 4.90; post 3.46 ± 5.26) IIQ score.

## 4. Discussion

To verify the goal of the work, we conducted a six-week high–low impact aerobics program supplemented with pelvic floor muscle training, preceded by a one-time EMG biofeedback session. The conducted experiment confirmed the hypothesis concerning statistically significant changes in the improvement of the technique of pelvic floor muscle exercises in physically active nulliparous women. Based on the available scientific data, this is the first study analyzing this issue. In order to achieve the assumed training effects, it is necessary to use a technique that allows us to learn and work out the correct technique of voluntary PFM contraction [28]. Isolating PFM to perform a voluntary contraction is a basic step to effective training, while being the most difficult task for a beginner. The ability to perform the correct PFMT technique is particularly important in future female exercise professionals [11]. Stephen et al., (2018) and Da Roza et al. (2012) demonstrated an increased exposure of fitness instructors and students of sports universities to urinary incontinence problems [29,30]. In our research, we checked the impact of a high–low impact exercise program supplemented with PFMT on the improvement of the PFM contraction technique of physically active women. The practical ability to use ’the knack’ reduces negative symptoms of the urinary tract, allowing these women to maintain a good quality of life [31].

The positive result is the statistically significant effectiveness of learning to contract the pelvic floor muscles after six weeks of training. A total of 100% of the intervention group in the post-intervention assessment properly activated PFM first in order in the quick flick contraction. In the control group, this task was performed correctly by 67% of participants.

A statistically significant improvement in the first and fifth 10-s contraction was also observed in the intervention group. In the post-interventional assessment in the first 10-s PFM contraction, 33% more of the control group performed the exercise with the correct technique. In the fifth 10-s contraction in the post-intervention assessment, 50% more women performed a contraction technically correctly. These results can be interpreted as a significant progress in improving the technique of performed exercises. In the remaining parameters, we did not notice a statistically significant improvement, however in all motor tasks, we noticed a positive trend in the group participating in the training program.

Guidelines for the correct exercise technique to improve pelvic floor function are inconsistent and require further research. In 2019, Martin-Rodriguez and Bø summarized the impact of abdominal hypopressive technique (AHT) exercise on improving PFM mainly performed via transversus abdominis (TrA) [32]. Some authors suggest the connection of AHT with PFM contractions [33]. In contrast, others emphasize that additional TrA contractions can increase intra-abdominal pressure, which has a negative effect on the pelvic floor [32].

A joint report by The International Urogynecological Association (IUGA) and International Continence Society) shows the importance of training based on various techniques of conscious activation and relaxation of PFM using sEMG [34]. The use of PFM contraction analysis, taking into account the order of muscle activation, made it possible to characterize the course of an individual’s PFM contractions. Research by Leitner et al. (2019) confirmed the 100% feasibility and excellent evaluation of sEMG signal processing in the analysis of fast voluntary PFM contractions [35]. Our research corresponded to the above. We assessed 42 participants with the sEMG, analyzing each of the seven motor tasks (which gave us 294 EMG records).

In a review of the literature on the effects of physical activity on the pelvic floor, Nygaard and Shaw highlight the problem of urinary incontinence in young girls during exercise based on jumping [36]. In a cross-sectional study by Bø et al. (2011), the level of UI symptoms was determined among female group fitness instructors. Among 685 fitness instructors (mean age = 32), 21.4% reported uncontrolled leakage of urine ≥ once a week [37]. Our research confirms that introducing PFM enhancement exercises into high-impact training reduces UI symptoms in young physically active women. The severity of urinary tract disorders is also aggravated by obesity, whereas participation in physical activity contributes to a reduction in the rate of overweight and obesity, which may be associated with preventing the development of UI [38]. The high–impact aerobics program, in which the training intensity increases by a combination of jumps, running and steps with different phases of flight, has a positive effect on the improvement in endurance parameters. The incorporation of PFMT into high-intensity activities prevents the deterioration of pelvic floor muscle function and maintains a good quality of life in relation to urinary incontinence [39]. Physically active women should not refrain from high-low intensity programs due to the fear of urine loss during exercise sessions. However, they should incorporate an important preventive element, which is the ability to correctly contract pelvic floor muscles in the moments of increased intra-abdominal pressure [39]. It is also worth emphasizing that our study group consisted of young women at reproductive age who are likely to become pregnant soon. According to the current World Health Organization recommendations, they can continue their pre-pregnancy activity, even if it was high-intensity activity [40]. The research available shows that it has many health benefits for mothers and babies [31]. In order not to have a negative effect on the functions of the pelvic floor muscles in pregnancy, preventive measures should be taught to women before pregnancy. The youth education on PFM should start as a part of physical education at schools.

## 5. Limitations

Owing to numerous speculations about the negative influence of high-impact physical activity on PFM function, we conducted this study as a pilot trial on a group of women presenting a good quality of life in terms of urinary incontinence. The above recommendations apply to the group of healthy nulliparous women. Further research is necessary to assess the effects of our intervention in women with symptoms of urinary incontinence. So far, it is not possible to promote our recommendations on high-low impact aerobics combined with PFMT for all women.

Another limitation is the substantial loss of study participants in the intervention group. It may be related to the requirements that we set for these women. The intervention group was supposed to participate in a training session three times a week for six weeks. The participants of the study were students of a sports university where sport practical classes were included in the study program. The loss might be due to having a reduced availability to actively participate in the training sessions, or due to having too much exercise during a week. Another weakness of our research is that we only used questionnaires to assess potential urinary incontinence. In the future, the assessment of the degree of dysfunction of the urinary system among the participants could be supplemented with a more reliable tool, e.g., a pad test.

## 6. Conclusions

The presented intervention was beneficial for the improvement of the PFM contraction technique and the observed changes were statistically significant. Supplementing high–low impact exercise programs with pelvic floor muscle training enables physically active nulliparous women to participate in various sports and recreational activities without causing undesirable changes in pelvic floor muscle function. Asymptomatic individuals should get education and instructions on how to implement PFM training. The EMG biofeedback sessions should also be promoted among women to teach them how to contract PFM correctly. However, due to the potential costs, they might be limited to women suffering from PFM dysfunctions.

## Figures and Tables

**Table 1:** Characteristics of the study participants.

Variable at Baseline	All Participants *n* = 42	Intervention Group *n* = 18	Control Group *n* = 24	*p*-Value
Age (years)	22 ± 2	22 ± 2	22 ± 2	0.15
BMI (kg∙m^−2^)	22.15 ± 2.82	21.15 ± 1.47	21.91 ± 3.52	0.60
Incontinence Impact Questionnaire (IIQ score)	3.29 ± 4.88	4.76 ± 4.90	2.18 ± 4.65	0.05
The firing order of the pelvic floor muscles in the following motor tasks *:				
quick flick	2.10 ± 1.78	2.33 ± 1.94	1.92 ± 1.67	0.60
first 10-s contractions	2.40 ± 1.89	2.56 ± 2.01	2.29 ± 1.83	0.81
second 10-s contractions	2.60 ± 1.96	2.56 ± 2.01	2.63 ± 1.97	0.96
third 10-s contractions	2.38 ± 1.79	2.56 ± 1.89	2.25 ± 1.75	0.66
fourth 10-s contractions	2.45 ± 1.88	2.39 ± 1.79	2.50 ± 1.98	0.98
fifth 10-s contractions	2.48 ± 1.85	2.94 ± 1.92	2.13 ± 1.75	0.15
60-s contraction	1.83 ± 1.51	1.83 ± 1.47	1.83 ± 1.57	0.96
Technique scores for pelvic floor muscle contraction in the following motor tasks **:				
quick flick	2.79 ± 1.41	2.72 ± 1.45	2.83 ± 1.40	0.96
first 10-s contractions	2.45 ± 1.37	2.56 ± 1.46	2.38 ± 1.31	0.49
second 10-s contractions	2.43 ± 1.47	2.56 ± 1.58	2.33 ± 1.40	0.50
third 10-s contractions	2.33 ± 1.37	2.17 ± 1.47	2.46 ± 1.32	0.56
fourth 10-s contractions	2.43 ± 1.47	2.44 ± 1.62	2.42 ± 1.38	0.85
fifth 10-s contractions	2.36 ± 1.45	2.06 ± 1.63	2.58 ± 1.28	0.37
60-s contraction	2.71 ± 0.86	2.78 ± 0.88	2.67 ± 0.87	0.77

Values are means ± SD. The statistical significance level was obtained using a Mann–Whitney test (the name of a variable in italics) or unpaired *t*-test (the name of a variable in normal font); *p* < 0.05 was considered statistically significant. IIQ score = item responses are assigned values of zero for “not at all,” one for “slightly,” two for “moderately,” and three for “greatly.” The average score of items responded to is calculated. The average, which was between zero and three, was multiplied by 33 1/3 to put scores on a scale of 0–100. * Scale firing order of the pelvic floor muscles: (1) PFM activated first; (2) PFM activated second; (3) PFM activated in third order; (4) PFM activated fourth in sequence; (5) lack of PFM neuromuscular activity. ** Score scale for the technique taking into account the isolation of PFM from synergistic muscles: four score—PFM contraction was recorded, maintaining the synergistic effect of relaxed muscles; three score—activation of PFM first, then selected synergistic muscles; two score—activation of synergistic muscles first, then PFM contraction; one score—activation of only synergistic muscles, leaving PFM muscles relaxed.

**Table 2:** The firing order and technique scores for pelvic-floor muscle contraction pre- and post- intervention.

Variable	Intervention Group (*n* = 18)		Control Group (*n* = 24)		ANOVA *p*-Value
	Pre-Intervention (mean ± SD)	Post-Intervention (mean ± SD)	Pre-Intervention (mean ± SD)	Post-Intervention (mean ± SD)	
**The firing order of the pelvic floor muscles in the following motor tasks:**					
quick flick	2.33 ± 1.94	1 ± 0 *	1.92 ± 1.67	2.25 ± 1.85	0.04
first 10-s contractions	2.56 ± 2.01	1.06 ± 0.24 *	2.29 ± 1.83	1.88 ± 1.65	0.04
second 10-s contractions	2.56 ± 2.01	1.5 ± 1.29	2.63 ± 1.97	2.46 ± 1.86	0.33
third 10-s contractions	2.56 ± 1.89	2.39 ± 1.82	2.25 ± 1.75	2.58 ± 1.93	0.65
fourth 10-s contractions	2.39 ± 1.79	2.33 ± 1.94	2.5 ± 1.98	2.17 ± 1.74	0.9
fifth 10-s contractions	2.94 ± 1.92	1.44 ± 1.29 *	2.13 ± 1.75	2.29 ± 1.83	0.04
60-s contraction	1.83 ± 1.47	1.17 ± 0.71	1.83 ± 1.58	1.63 ± 1.31	0.39
**Technique scores for pelvic-floor muscle contraction in the following motor tasks:**					
quick flick	2.72 ± 1.45	3.61 ± 0.5 *	2.83 ± 1.4	2.58 ± 1.53	0.03
first 10-s contractions	2.56 ± 1.46	3.22 ± 0.55	2.38 ± 1.31	2.75 ± 1.36	0.2
second 10-s contractions	2.56 ± 1.58	2.89 ± 1.02	2.33 ± 1.4	2.25 ± 1.59	0.83
third 10-s contractions	2.17 ± 1.47	2.17 ± 1.25	2.46 ± 1.32	2.21 ± 1.53	0.44
fourth 10-s contractions	2.44 ± 1.62	2.28 ± 1.49	2.42 ± 1.38	2.46 ± 1.5	0.96
fifth 10-s contractions	2.06 ± 1.63	2.72 ± 1.02	2.58 ± 1.28	2.42 ± 1.44	0.67
60-s contraction	2.78 ± 0.88	3.06 ± 0.42	2.67 ± 0.87	2.88 ± 0.95	0.65

Values are means ± SD; Friedman ANOVA test; Dunn–Bonferroni post-hoc tests; *p* < 0.05 was considered statistically significant *. Scale firing order of the pelvic floor muscles: (1) PFM activated first; (2) PFM activated second; (3) PFM activated in third order; (4) PFM activated fourth in sequence; (5) lack of PFM neuromuscular activity. Score scale for the technique taking into account the isolation of PFM from synergistic muscles: 4 score—PFM contraction was recorded, maintaining the synergistic effect of relaxed muscles; three score—activation of PFM first, then selected synergistic muscles; two score—activation of synergistic muscles first, then PFM contraction; one score—activation of only synergistic muscles, leaving PFM muscles relaxed.

## Data Availability

The data that support the findings of this study are available from the corresponding author upon reasonable request.
